# Validation of the short assessment of health literacy (SAHL-D) and short-form development: Rasch analysis

**DOI:** 10.1186/s12874-019-0762-4

**Published:** 2019-06-14

**Authors:** A. J. Woudstra, C. S. Meppelink, H. Pander Maat, J. Oosterhaven, M. P. Fransen, A. L. Dima

**Affiliations:** 1 Department of Public Health, Amsterdam Public Health research institute, Academic Medical Center, University of Amsterdam, Amsterdam, The Netherlands; 2 Amsterdam School of Communication Research, University of Amsterdam, Amsterdam, The Netherlands; 3 Utrecht University of Linguistics OTS, Utrecht University, Utrecht, The Netherlands; 4 Research Group Lifestyle and Health, University of Applied Sciences Utrecht, Utrecht, 3584 CS The Netherlands; 5 Health Services and Performance Research (HESPER EA 7425), University Claude Bernard Lyon 1, Lyon, France

**Keywords:** Health literacy, Measurement, Public health, Rasch, SAHL-D, Short-form

## Abstract

**Background:**

Accurate measurement of health literacy is essential to improve accessibility and effectiveness of health care and prevention. One measure frequently applied in international research is the Short Assessment of Health Literacy (SAHL). While the Dutch SAHL (SAHL-D) has proven to be valid and reliable, its administration is time consuming and burdensome for participants. Our aim was to further validate, strengthen and shorten the SAHL-D using Rasch analysis.

**Methods:**

Available cross-sectional SAHL-D data was used from adult samples (*N* = 1231) to assess unidimensionality, local independence, item fit, person fit, item hierarchy, scale targeting, precision (person reliability and person separation), and presence of differential item functioning (DIF) depending on age, gender, education and study sample.

**Results:**

Thirteen items for a short form were selected based on item fit and DIF, and scale properties were compared between the two forms. The long form had several items with DIF for age, gender, educational level and study sample. Both forms showed lower measurement precision at higher health literacy levels.

**Conclusions:**

The findings support the validity and reliability of the SAHL-D for the long form and the short form, which can be used for a rapid assessment of health literacy in research and clinical practice.

**Electronic supplementary material:**

The online version of this article (10.1186/s12874-019-0762-4) contains supplementary material, which is available to authorized users.

Health literacy is essential for access to health care and services, informed decision making about health and self-management of disease. Health literacy is defined as ‘the ability to obtain, process, and understand basic health information and services needed to make appropriate health decisions’ [1]. Previous studies showed that low health literacy is associated with poorer health outcomes, poorer use of health care services and poorer health-related knowledge and comprehension [2]. For instance, people with low health literacy are often more often chronically ill, hospitalized, and participate less often in preventive care programmes compared to people with adequate health literacy levels [2–4].

Low health literacy is a growing public health concern. Results from the recent European Health Literacy Survey (HLS-EU) showed a very high prevalence of inadequate health literacy across European countries. At least 1 in 10 (12%) of the participants had insufficient health literacy and almost 1 in 2 (47%) had limited (insufficient or problematic) health literacy [5]. According to the 2003 National Assessment of Adult Literacy (NAAL), more than one third (36%) of the adult population in the United Stated had basic or below health literacy skills [6].

To ensure equal access to health care for those with low health literacy, accurate and efficient health literacy measurement is needed [7]. Over the past decade, more than 150 health literacy measures have been developed worldwide. There is a wide variety of health literacy measures in different languages, ranging from more general measures to context-specific ones, and from performance-based to self-reported measures [8]. The procedures to validate health literacy measures are mostly guided by classical test theory (CTT) approaches. However, recently, several authors have increasingly expressed the need for modern measurement approaches to health literacy scale development, such as item response theory (IRT) and Rasch modeling [8–10].

One measure frequently applied in international research is the Short Assessment of Health Literacy (SAHL). This performance-based 33-item measure has proven valid and reliable and was previously adapted for use in the Netherlands (SAHL-D) [11]. The original SAHL-D has previously been analyzed using CTT approaches, showing preliminary validation results such as good internal consistency (Cronbach’s alpha = 0.77 for recognition, 0.79 for comprehension and 0.86 for the total score) [11]. However, in the validation study of the original SAHL-D, item properties were not further explored [11]. In addition, although the original SAHL-D has shown to be a valuable tool for research and the development of HL interventions, administration is complex and time consuming due to its length and personal face-to-face administration. Finally, the SAHL-D validation was performed among selected samples (students and an online research panel) that were likely to have had higher levels of health literacy compared to the general Dutch population.

Compared with CTT, IRT approaches have several advantages to develop and validate health literacy measures [9]. First, IRT analysis provides the opportunity to examine the level of health literacy that is being measured (e.g. low or adequate) and where new items should be developed (e.g. there may be a need for the development of easier or more difficult items). Second, IRT analyses may improve measurement precision by producing items statistics that are independent of item or person statistics [12]. In essence, IRT investigates the extent to which an item set meets several criteria necessary for precise measurement.

One of the strictest and most parsimonious IRT models for dichotomous response formats is the Rasch model [13]. Rasch analysis allows researchers to use test-takers’ original test scores and express the test-takers’ performance on a linear scale that accounts for the unequal difficulties across all test items, in contrast to CTT which assumes that all test items have equal difficulty levels. For instance, when items are intended to be summed together to provide a total score, Rasch analysis involves corrections for a number of psychometric issues so that accurate person measures can be calculated [14, 15].

If a set of health literacy items meets the Rasch criteria, respondents’ answers can be used to calculate the precise location of each respondent on a latent continuum from low to high health literacy, i.e. their ‘person ability’ levels. Moreover, items themselves can be located on this dimension according to their ‘item difficulty’ levels, i.e. the location on this latent continuum at which an item has 50% probability of being answered correctly. By selecting only items that fit the Rasch model, the measurement precision, or reliability, is enhanced [15]. Third, an underlying assumption of Rasch models is that the estimated item parameter values should be similar for different groups. When estimated item parameters behave differently across groups after controlling for ability, an item is considered to have differential item functioning (DIF). Rasch analyses can help identify items with DIF that may be revised or omitted [8].

Given these theoretical considerations, two practical applications of Rasch analyses can be considered for further improvement of the SAHL-D. First, item difficulty parameters can be used to calibrate items on a common difficulty scale for computerized adaptive testing (CAT). Consequently, CAT enables tailoring test administration to the ‘ability’ of each respondent by adjusting the sequence of displayed items based on the respondent’s previous answers. This results in administering fewer items per respondent from the larger item bank of the original test [16], thus reducing administration time and respondent burden. This also provides opportunities for health care providers and patients to receive test results and use this information instantly to inform medical decision making [7]. Establishing an item bank can also help address the increasing amount of health literacy measures, the lack of standardization in health literacy measurement and enable the comparison of scores across studies and populations [8].

Second, for clinical or research settings where administration time needs to be reduced, yet no resources are available for CAT, Rasch item diagnostics can be used to develop a short form of the scale by carefully selecting best fitting items that maintain the scale properties within optimal parameters. Therefore, to improve this measure, this study aimed to perform comprehensive Rasch analyses of the SAHL-D to (1) calibrate items for CAT and (2) subsequently develop a short form.

## Method

### Health literacy measure: SAHL-D

The SAHL-D contains 33 items consisting of single words that refer to medical specialties, tests, treatments and symptoms [11]. People have to pronounce each word of the test which has to be rated by a coder as either correct or incorrect. Additionally, people have to select the correct meaning of each word, using a multiple-choice format with one correct response option, two distractor options, and an ‘I don’t know’ option. One point is given for the correct meaning (comprehension test) and one point is given for the correct pronunciation (recognition test). Consequently, health literacy scores range from 0 to 66.

### Participants

The validity of the SAHL-D was analyzed among four adult samples (*N* = 1231). Analyses were performed on the comprehension part of the SAHL-D, and not on the recognition part (i.e. pronunciation of words), because the former is more relevant for online self-administered questionnaires as opposed to administered face-to-face questionnaires. In addition, medical words may be pronounced correctly, without being understood [8].

The participants in the first two samples were recruited by an International Organization for Standardization (ISO) certified European online recruitment agency [17]. The first sample (*n* = 559) included healthy Dutch adults over the age of 55 [18]. The demographic characteristics of the second sample (*n* = 231) were similar to the first sample (healthy Dutch adults over age 55 years) [19]. The third sample (*n* = 329) was derived from the SAHL-D validation study. The participants from the SAHL-D validation study were healthy adults between the ages of 20–85 years old and were recruited by a test panel of the Netherlands Institute for Health Service Research (NIVEL) [11]. The fourth sample (*n* = 112) included patients with chronic musculoskeletal pain (ages 17–76 years old) [20] and was recruited in an interdisciplinary outpatient rehabilitation center (Heliomare Wijk aan Zee) in the Netherlands. All data were collected between 2013 and 2015. Patients unable to understand the Dutch written language were excluded in all four samples.

Educational levels were classified into low, middle and high following the International Standard Classification of Education (ISCED); low (level 0–2: early childhood; primary education, lower secondary education); intermediate (level 3–5: upper secondary, post secondary, short cycle tertiary) and high (level 6–8: bachelor, master, doctoral) [21]. To test whether item difficulty scores were independent of age, the variable age was dichotomized (first group < 65 years; second group ≥65 years) based on the median, evidence of cognitive aging and lower health literacy above the age of 65 [22, 23].

### Data analysis

Rasch analyses were performed in WINSTEPS [24]. Descriptive statistics were performed for sample characteristics, such as age, gender and educational level using SPSS version 24. The psychometric analysis consisted of two steps. First, we examined the psychometric properties of the 33-item SAHL-D. Second, the scale was shortened by selecting the best performing items. Although the scale gives four response options among which only one is correct, most of the data available (samples 1, 3 and 4) had been recorded as binary variables (correct versus incorrect response); we therefore used the Rasch model for binary data to perform these analyses [14]. For the long form, a distractor analysis was performed in one sample where all response options were recorded (sample 2).

### Step 1. Validation and item calibration of the original form SAHL-D

We followed recommendations on Rasch modeling [14], measurement of health literacy [25] and previously published Rasch analyses of an eHealth Literacy scale [10, 26]. To assess dimensionality, we examined the variance explained by the first contrast in the residuals by performing a principal components analysis (PCA) on the standardized residuals. Item fit and person fit was determined using mean square fit statistics; infit and outfit values between 0.6 and 1.4 were considered to indicate appropriate fit, while higher and lower values indicate under- and overfit, respectively (i.e. the data were less or more predictable than the model expects).

The acceptable range for standardized *t* scores (represented as ZSTD in Winsteps) is ±2.0 on a low stakes test. The ZSTDs represent the significance of the misfit [27]. To examine whether the items have comparable difficulty in all demographic groups, we examined the presence of differential item functioning (DIF) depending on age, gender, education and study sample. Item difficulty is expressed in logits (log-odds units), which represent the odds ratio of an item being answered incorrectly versus correctly, scaled as natural logarithm to allow comparison of items and respondents. A value of 0 logit represents ‘medium difficulty’ (50% probability of correct response by a respondent of medium ability) and negative or positive values stand for lower or higher difficulty [28]. A criterion of at least .5 logit difference with a *p* value < 0.05 (according to the Rasch-Welch test) was used for detecting DIF [29].

The reliability of the SAHL-D items was examined by the index of person separation, which is similar to Cronbach’s alpha. Person separation is used to classify people and estimates how well a measure can separate individuals on a construct. High person separation or strata (≥ 2, representing two different levels of performance, i.e. high and low, that can be distinguished based on test scores, person reliability ≥0.7) implies that the measure may be sensitive to distinguish between high and low performers. Item separation is used to verify the item hierarchy. High item separation or strata (≥ 3, representing three different levels of difficulty, i.e. high, medium, and low; item reliability ≥0.9) implies that the person sample is large enough to confirm the item difficulty hierarchy [30].

A distractor analysis for sample 2 (the only sample with recorded multiple response options) was performed to obtain more information on the behavior of the incorrect response options. According to Rasch model assumptions, the respondents who select the correct option should have higher average ability levels than respondents who select the other options. We would also expect that the ‘I don’t know’ option would be selected by respondents with lower ability levels, unless one of the distractors would be a stronger signal of low health literacy. Importantly, each response option should have a minimum of 10 observations to justify its selection among the available options [30].

### Step 2. Short form of SAHL-D

Item selection was performed based on two criteria. First, we examined items with infit and outfit mean squares outside the 0.6 to 1.4 range and standardized fit statistics outside the +/− 2.0 range [27]. Second, DIF was examined in relation to gender, age, education and study sample; the threshold for exclusion was a noticeable and significant difference of at least .5 logits [31] (see Additional file 1 for item difficulty parameters per demographic characteristic). Items were excluded one by one and parameters were re-computed for the remaining items, while monitoring the resulting overall item level fit. Scale targeting and person reliability were examined after each deletion to ensure that the remaining item set is well distributed across different levels of the latent continuum, and that reliability remains acceptable. Item difficulty parameters, person fit, scale targeting and person reliability and separation were calculated for the resulting short form and compared to the original long form.

To determine optimal cut-off scores for the short SAHL-D, we calculated receiver operating characteristic (ROC) curves using SPSS Inc. Version 24.0 [32]. To calculate the ROC curves, we used adequate prose literacy as the reference standard, which was assessed in the original SAHL-D validation study [11]. Prose literacy was assessed by a subset of items from a reading comprehension test widely used for ninth graders in Dutch pre-university secondary education (total 16 items) (*n* = 222). The prose literacy test and the cut-off values (scores ≤6 reflect inadequate prose literacy; scores ≥7 reflect adequate prose literacy) are described in more detail elsewhere [11].

## Results

Of the respondents in the whole sample (*N* = 1231), 48.8% were male (*n* = 601). The mean age was 62.7 years (*SD* = 12.7). The mean SAHL-D score was 24.4 (*SD* = 6.3). More than 20% of the respondents (*n* = 269) had lower education, 33.5% (*n* = 412) had middle education and 43.5% (*n* = 536) had higher education. About 1.1% (*n* = 14) had missing values for education (see Table 1).

**Table 1:** Demographic characteristics of four study samples (*N* = 1231)

	Sample 1 (*n* = 559)	Sample 2 (*n* = 231)	Sample 3 (*n* = 329)	Sample 4 (*n* = 112)
Sex	*n* (%)	*n* (%)	*n* (%)	*n* (%)
Male	313 (56)	121 (52.4)	136 (41.3)	31 (27.7)
Female	246 (44)	110 (47.6)	193 (58.7)	81 (72.3)
Age (mean, SD)	67.2 (2.3)	68.2 (2.1)	56.2 (2.3)	48.3 (2.2)
Education				
Lower	139 (24.9)	96 (41.6)	26 (7.9)	8 (7.1)
Middle	138 (24.7)	27 (11.7)	189 (57.4)	68 (60.7)
High	282 (50.4)	108 (46.8)	110 (33.4)	26 (23.2)
Missing	0	0	4 (1.2)	10 (8.9)
SAHL-D score (mean, SD)	24.2 (6.6)	23.3 (7.4)	26.4 (4.3)	22.4 (6.0)

### Results step 1. Validation and calibration of the original form SAHL-D

#### Dimensionality detection

A PCA of the Rasch model standardized residuals indicated that the Rasch model explained 36.3% of the variance. The remaining variance did not form additional dimensions with eigenvalues < 2.0. This result indicates that the SAHL-D meets Rasch criteria of unidimensionality and local independence [31]. For all items, respondents with higher health literacy levels were more likely to choose the correct response option; thus, all items showed the expected positive associations with the latent dimension.

#### Item fit

Table 2 displays the item difficulty, infit and outfit parameters. Infit mean squares ranged from 0.81 to 1.17 and outfit mean squares ranged from 0.42 to 1.88. Thus, for infit statistics, all items fit the model. For outfit statistics, two items *Adrenalin* and *Beta blocker* showed underfit (> 1.4) and three items *Spinal cord lesion*, *Oncology* and *Defibrillation* showed overfit (< 0.6) *Adrenalin* had the highest outfit because this rather difficult item (1.88 logits) was answered incorrectly by respondents who scored middle to high on the latent continuum. *Beta blocker* was answered correctly by respondents with low overall health literacy scores (see Additional file 2 for item difficulty parameters per study sample).

**Table 2:** Item fit in order of difficulty *(N* = 1231)

Item	Measure	Model	Infit		Outfit	
		S.E.	MNSQ	ZTSD	MNS	ZSTD
Ventricle	2.95	.07	.96	−1	.89	− 1.3
Manic	2.2	.07	.99	−0.4	1.14	1.9
Reflux	1.85	.07	.92	−2.9	.97	− 0.4
Gelling agent	1.81	.07	1.14	5.1	1.20	3.1
Palliation	1.55	.07	.86	−5.6	1.12	1.9
Hemophilia	1.41	.07	1.05	1.7	1.05	0.9
Pessary	1.27	.07	.99	−0.4	.95	−0.8
Malaise	1.05	.07	1.13	4.2	1.23	3.4
Orthodontia	0.99	.07	1.17	5.1	1.25	3.7
Beta-blocker	0.87	.07	1.04	1.3	1.53	7
Chlamydia	0.81	.07	.99	−0.3	.98	−0.3
Prenatal	0.74	.07	.90	−3	.83	−2.7
Resistance	0.7	.07	1.15	4.3	1.26	3.5
Pancreas	0.61	.07	.85	−4.5	.74	− 4.1
Echography	0.6	.07	1.09	2.6	1.11	1.6
Apathy	0.36	.08	.96	−1	.90	−1.3
Chiropractor	0.14	.08	1.12	2.8	1.31	3.3
Delirium	0.02	.08	1.11	2.4	1.12	1.3
Psoriasis	0	.08	1.05	1.1	1.14	1.5
Edema	−0.35	.09	.89	−2.1	.73	−2.6
Hospice	−0.54	.09	.95	−0.9	.91	− 0.7
Biopsy	−0.75	.10	.91	−1.3	.77	− 1.8
Euphoria	−0.83	.10	.90	−1.5	.63	−2.9
Oncology	−0.95	.10	.90	−1.4	.54	−3.6
Plaque	−1.12	.11	.81	−2.6	.60	− 2.7
Obesity	−1.13	.11	.96	−0.5	.96	−0.2
Flaking	−1.15	.11	.99	−0.1	1.22	1.3
Apnea	−1.25	.11	1.05	0.6	.93	−0.3
Schizophrenia	−1.32	.11	.94	−0.7	.86	−0.7
Adrenalin	−1.89	.14	1.05	0.5	1.88	3.1
Achilles tendon	−2.44	.17	.96	−0.3	.70	−1.1
Defibrillation	−2.92	.20	.85	−0.9	.42	−2.4
Spinal cord lesion	−3.27	.22	.90	−0.5	.56	−1.5

#### Person reliability and separation

The person reliability was .83 and person separation was 2.22, indicating that the SAHL-D separated the sample into 3.2 strata. This means that SAHL-D is able to distinguish statistically between three groups (high, middle, and low performers) [30].

#### DIF (age, gender, education and study sample)

##### Age DIF

For age, six items showed significant DIF (at least .50 logits; *p* < 0.05). For the older age group (≥ 65 years), *Apathy (1.08; p < .001)* and *Edema (.83; p < .001)* were easier. In contrast, four items *Orthodontia (−.53; p < .001), Schizophrenia (−.63; p = .007), Obesity (−.52; p = .019)* and *Apnea (−.57; p = .013)* were easier for the younger age group (< 65 years).

##### Gender DIF

Four items displayed significant DIF (at least 50 logits; *p* < 0.05) for gender. For males, the items *Euphoria (−.85; p < .001)* and *Resistant (−.92); p < .001)* were easier. For females, the items *Hospice (67; p < .001) and Reflux (.53, p < .001)* were easier.

##### Education DIF

Table 3 displays significant DIF (at least .5 logits; *p* < 0.05) among the three educational groups (low, middle, high). Four items *Euphoria, Pancreas, Prenatal, Palliation* were easier for those with high educational level compared to those with middle or low educational level. *Euphoria* and *Pancreas* were easier for those with middle educational compared to those with low educational level. In contrast, six items *Apnea, Psoriasis, Adrenalin, Malaise, Delirium* and *Chlamydia* were easier for those with low educational level compared to those with high educational level. *Psoriasis* and *Chlamydia* were easier for those with middle educational level compared to those with high educational level. Two items *Delirium* and *Apnea* were easier for those with low educational level compared to those with middle educational level.

**Table 3:** DIF for educational groups (*N =* 1217)

Item	Educational group^a^	DIF contrast^b^	Prob (Welch t)^c^
Apnea	1 vs 3	−1.01	.0003
Psoriasis	1 vs 3	−.70	.0005
Adrenalin	1 vs 3	−.67	.8777
Malaise	1 vs 3	−.63	.0005
Delirium	1 vs 3	−.53	.0128
Chlamydia	1 vs 3	−.50	.0059
Psoriasis	2 vs 3	−.72	.0001
Chlamydia	2 vs 3	−.60	.0002
Delirium	1 vs 2	−.70	.0007
Apnea	1 vs 2	−.62	.0260
Palliation	3 vs 2	−.74	.0000
Prenatal	3 vs 2	−.76	.0000
Euphoria	2 vs 1	−.75	.0014
Pancreas	2 vs 1	−.59	.0015
Euphoria	3 vs 1	−1.17	.0000
Pancreas	3 vs 1	−.91	.0000
Prenatal	3 vs 1	−.96	.0000
Palliation	3 vs 1	−.53	.0044

^a^Note. Education group 1 = low; 2 = middle; 3 = high

^b^Note. The DIFcontrast measure illustrates the difference between the item measures or the difference of the item between two groups. A difference of at least 0.50 logits is required for DIF to be noticeable. A negative DIF contrast means that the item was easier for the group on the left, whereas a positive DIF contrast means that the item was more difficult for the group on the left

^c^Note. The Prob illustrates the probability of observing this amount of contrast by chance. Only items that are statistically significant at the *p* < 0.05 level are displayed

##### Study sample DIF

Of the 33 items, 18 items (*Adrenalin, Apathy, Apnea, Chiropractor, Chlamydia, Defibrillation, Delirium, Echography, Edema, Euphoria, Flaking, Gelling agent, Manic, Orthodontia, Palliation, Reflux, Resistance and Ventricle)* displayed significant DIF (at least .5 logits; *p* < 0.05) between the four samples (see Additional file 3 for a complete table of DIF per sample. Only items that are statistically significant at the *p* < 0.05 level are shown).

#### Scale targeting

**Fig. 1:**
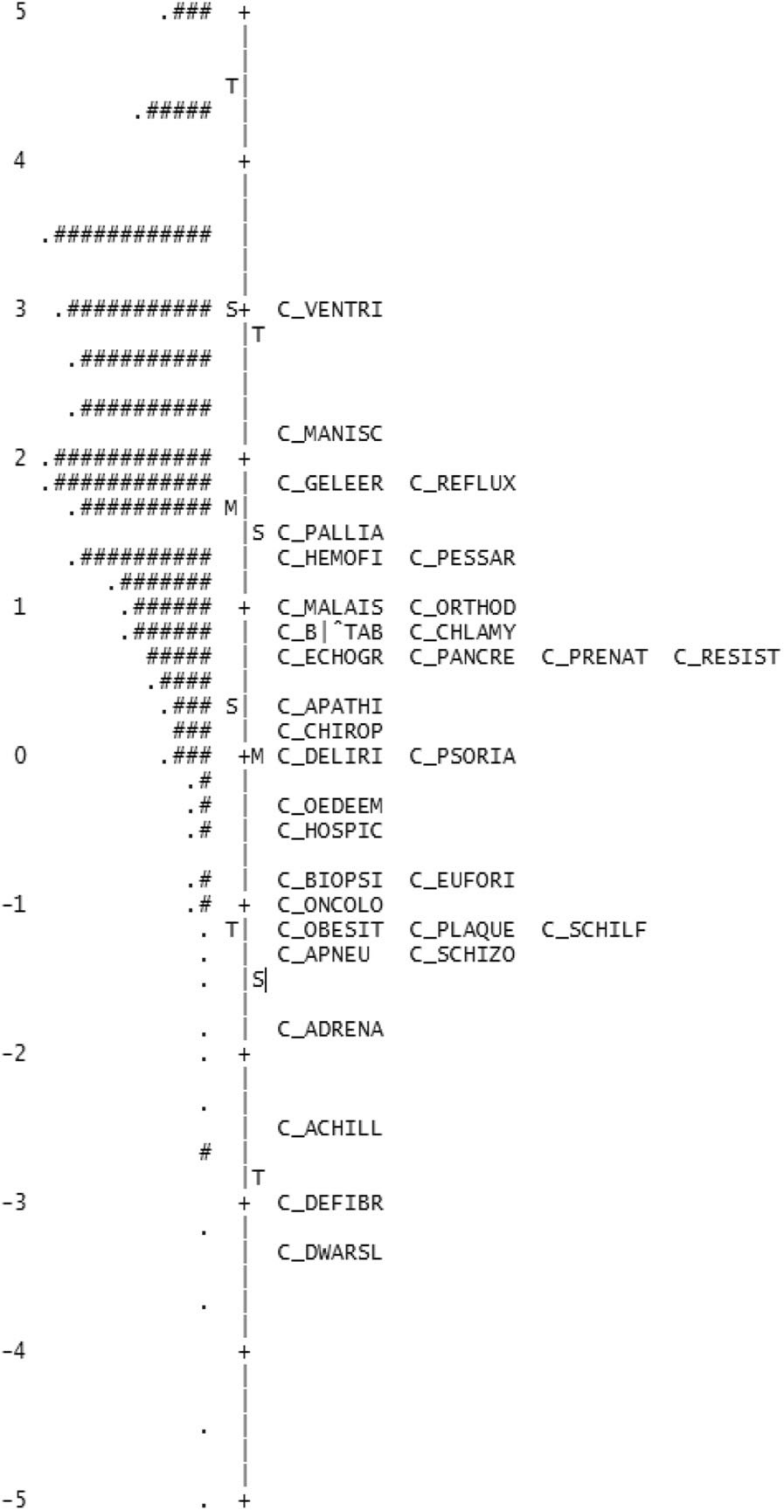
Person ability item-difficulty match (*N* = 1231). Person ability distribution is shown on the left of the vertical line. Each “#” is 9 persons; each “.” is 1 to 8 persons

Figure 1 locates both items (difficulty levels) and persons (distribution of person ability scores) on the same continuum of health literacy. The distribution of person ability scores is shown on the left and item difficulty values are shown on the right side of the line, from easiest/less able (bottom) to most difficult/more able (top). The higher the person measure, the better their performance. The figure indicates that the SAHL-D items were relatively evenly distributed across levels of health literacy from − 3 to + 3 logits. However, a substantial part of the sample investigated had higher levels of health literacy (> 2 logits) and only one item (*Ventricle*) was located in this area of the latent continuum, suggesting that the current form of the test is less able to differentiate between respondents with high levels of health literacy. In contrast, the lower end of the scale includes several items that might be redundant for the samples tested, as very few respondents have low health literacy levels (≤ 1 logits) and several items measure the same level of the construct.

#### Distractor analysis

An analysis of individual response categories using the original multiple-choice format from sample 2 showed that the correct options were most frequently chosen by high health literate respondents over all other options, and the ‘I don’t know’ options were systematically the most common choice among the low health literate respondents; for 3 items, one distractor option had lower score than the ‘don’t know’ option (option 2 for item *Ventricle,* option 1 for item *Palliation*, and option 2 for item *Oncology* also had low performance (< 10 observations). Such low performance was also found in other distractors from 26 items, some of these response options had not been selected by any respondent. The easiest 5 items in this sample had both distractors with < 10 observations [33].

### Step 2: selecting best performing items for the short form of the SAHL-D

To arrive at a short form of the SAHL-D, we used several criteria for item exclusion. First, we examined item fit against the criteria of < 0.6 or > 1.4 and ≤ 2.0 or > 2.0 ZTSD. All infit and outfit values were within these limits. Second, based on previous recommendations for health literacy measurement [25], we excluded a total number of 19 items based on DIF ≥ .5 logits for age, gender, and educational level. DIF for study sample was not selected as a criterion for further item deletion, as this DIF had no clear distinct demographic characteristic across the study samples. For the development of comparable tests, it is essential to examine how the relationship between items and health literacy differs across different demographic groups and whether these differences reflect actual differences and not item bias [28].

We monitored scale targeting and person reliability after each item removal. Three items (*Resistant*, *Euphoria* and *Hospice*) were excluded based on DIF for gender. Four items (*Apathy, Edema, Schizophrenia* and *Hemophilia*) based on DIF for age, and 12 items (*Prenatal, Pancreas, Defibrillation, Apnea, Palliation, Achilles tendon, Oncology, Manic, Delirium, Spinal cord lesion, Adrenalin* and *Psoriasis*) based on DIF for educational level. We stopped item removal when all remaining items showed fit and DIF statistics within the set thresholds. The final item set (*n* = 13) had person reliability of .66 and person separation of 1.40, person fit (< 5% of the sample outside the fit range according to the same thresholds as item fit), and scale targeting (examined visually).

**Fig. 2:**
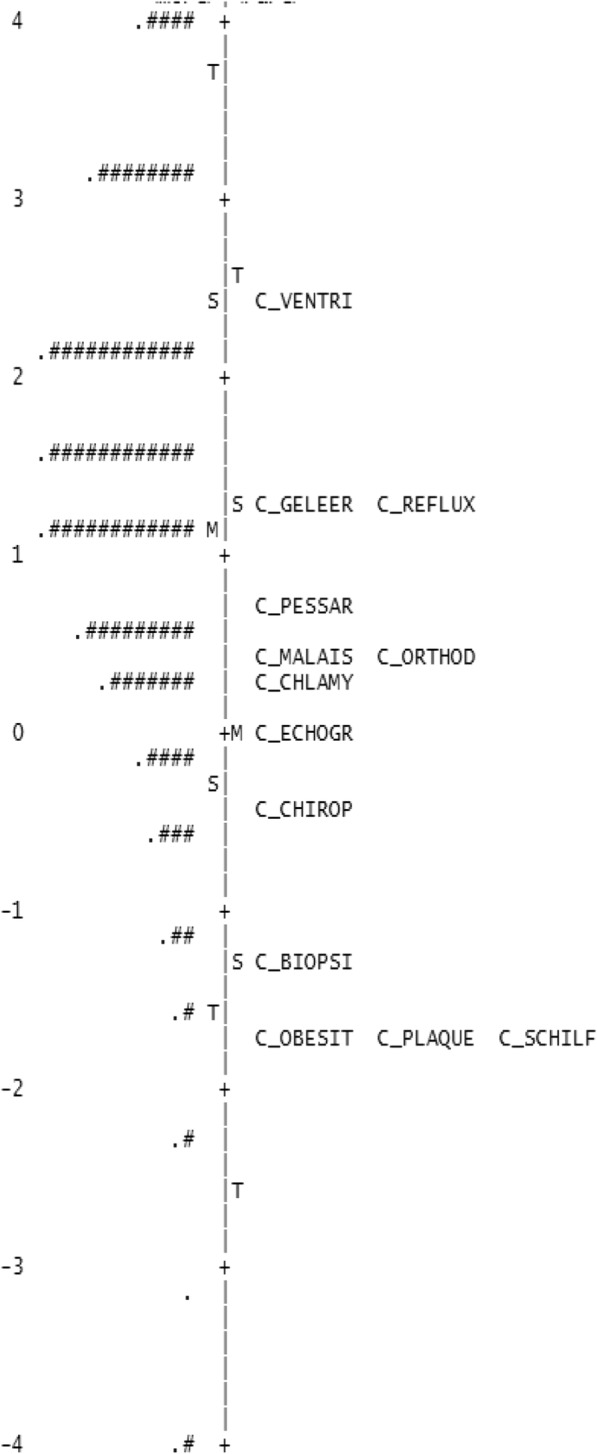
SHORT-FORM Person ability item-difficulty match (*N* = 1231). Person ability distribution is shown on the left of the vertical line. Each “#” is 9 persons; each “.” is 1 to 8 persons

Table 4 shows item statistics for the resulting SAHL-D short form. Figure 2 locates both items (difficulty levels) and persons (distribution of person ability scores) on the same continuum of health literacy. The figure indicates that some test items have similar difficulty levels (e.g. items *Gelling agent* and *Reflux*), yet most items target different health literacy levels.

**Table 4:** Short form item properties (*n* = 13) in order of item difficulty

Item	Measure	Model	Infit		Outfit	
		SE	MNSQ	ZSTD	MNSQ	ZSTD
Ventricle	2.47	0.07	0.95	−1.5	0.84	−1.8
Reflux	1.32	0.07	0.88	−4.6	0.86	−2.7
Gelling agent	1.29	0.07	1.11	3.8	1.17	3.1
Pessary	0.73	0.07	0.95	−1.8	0.88	−2.4
Malaise	0.5	0.07	1.13	4.2	1.24	4.2
Orthodontia	0.44	0.07	1.09	2.7	1.18	3.2
Chlamydia	0.25	0.07	0.93	−2	0.89	−1.9
Echography	0.04	0.07	1.04	1.1	1.01	0.1
Chiropractor	−0.44	0.08	1.1	2.3	1.29	3.3
Biopsy	−1.36	0.1	0.91	−1.4	0.78	−1.7
Plaque	−1.74	0.11	0.81	−2.7	0.7	−2
Obesity	−1.75	0.11	0.94	−0.7	0.9	−0.6
Flaking	−1.77	0.11	1.01	0.2	1.3	1.7

The area under the ROC curve was 0.73 (CI 0.65–0.82) for the short SAHL-D.

For the short SAHL-D, a cut-off score of 7.5 would correctly classify 38% of the test-takers with low HL and 92% of the test-takers with adequate HL. A cut-off score of 8.5 would correctly classify 58% of the test takers with low HL and 86% with adequate HL. A cut-off score of 9.5 would correctly classify 68% of the test-takers with low HL and 69% of the test-takers with adequate HL. Deciding on a final cutoff score depends on the use of the measure and the priorities in a given setting.

## Discussion

The aims of this study were to (1) calibrate items of the health literacy measure SAHL-D for CAT and (2) subsequently to select the best performing items for a short form. This is the first study to provide a thorough analysis of the SAHL-D using Rasch analysis. We offer concrete conclusions for applying the SAHL-D in more time-constrained settings. Both the long form and the short form had acceptable targeting and good reliability. Depending on the availability of technical solutions in a specific context, researchers or clinicians can use the long form to apply CAT or use the short form.

The scale has proven to be unidimensional, which implies that only one trait (health literacy) is being measured by the SAHL-D. All items had good fit within the thresholds stipulated (i.e. < 0.6 or > 1.4 and ≤ 2.0 or > 2.0 ZTSD). This means that the items at the more difficult end of the latent continuum were harder to correctly answer than the items at the easier end of the continuum. The findings showed a substantial variation in item difficulty which supports theories that consider health literacy as an ability [34]. Based on these results, we concluded that SAHL-D has good psychometric properties. The most difficult item was *Ventricle*, with a difficulty level of 2.95 logits. The easiest item was *Spinal cord lesion,* with a difficulty level of − 3.27 logits. We found relatively large differences in how easy or how difficult the items were for these respondents, yet the current scale includes a high number of relatively easy items. These results suggest that the scale targets medium level of health literacy, and has fewer items of higher difficulty.

The distractor analysis of study 2 showed that response options behaved mostly in the expected direction, as the correct answers were selected by more skilled respondents and the selection of ‘don’t know’ was an indicator of lower health literacy. The incorrect response options were shown to be less plausible than the correct options for the respondents of study 2. This may be due to the content of the incorrect option, the high plausibility of the correct option (also suggested by the low difficulty level of some items with less endorsed distractors), the low sample size for this separate analysis, and also as a result of the high levels of health literacy in this particular sample. This additional information can guide the development of new items or new item forms using different (more plausible) response categories for expanding the SAHL-D item bank. For example, one may consider replacing an obviously wrong distractor with a more plausible one.

A key advantage of Rasch modeling is that it can be used to examine whether test-takers who have approximately the same health literacy level, perform in a similar way on the individual test items across demographic groups. To the best of our knowledge, this is the first study that examines at an item level how age, gender, educational level and study sample may influence health literacy scores as measured by the SAHL-D. Of the 33 items, significant DIF occurred in 6 items for age, 4 items for gender, 10 items for educational level and 23 items for study sample, independent of the actual level of health literacy.

Prior research on health literacy measurement showed mixed results with regard to age-related DIF. While research on health literacy measurement using the Test of Functional Health Literacy (TOFHLA) [23] and the Newest Vital Sign (NVS) found age-related DIF [35], a study using the Rapid Estimate of Adult Literacy in Medicine (REALM) did not [36]. The different response formats (e.g. multiple choice, close response format) of these health literacy measures may be related to these age-related differences in health literacy. Ownby, Acevedo [23] found DIF for age using the TOFHLA (cloze response format) and recommend caution when selecting a health literacy measure with such response format for older adults.

A recent study on the development of a Functional Health Literacy Scale among Japanese young adults [37] also found items with DIF by gender. These differences in gender were explained by the fact that some items may be easier for the group with a higher incidence of a certain disease or more familiarity with certain preventive health behaviors. In our study, we found that two items (*Hospice and Reflux)* were easier for women and two items (*Euphoria* and *Resistant)* were easier for men, which may also be explained by more familiarity with certain health care situations. However, this would need further examination.

Although the individuals with higher educational level in this study were more likely to have higher health literacy levels compared to those with lower educational level, our aim was not to examine the association between educational level and health literacy, but rather how to measure an individual’s health literacy without bias for education. This is in line with previous recommendations on the measurement of health literacy [25]. Moreover, prior research has shown that health literacy is not necessarily related to years of education or functional literacy (i.e. reading and writing ability) [2] and that health literacy is a stronger predictor for an individual’s health status than educational level [38].

DIF for study sample was also found in previous research on the construct validity of the eHealth Literacy Scale among two adult populations by Nguyen et al. [26], who found that the difficulty order of the items depends on the study sample. The findings related to sample-related DIF, together with those of previous studies on different health literacy measures, suggests that testing differences in item difficulty among different populations (e.g. healthy versus suffering from specific medical conditions) may prove useful for understanding the concept of health literacy, as measured by various performance-based or self-reported measures. The fact that various objective or subjective indicators of health literacy may behave differently depending on respondents’ experiences of health and illness may represent a characteristic of health literacy worth exploring further, and not a drawback of individual items.

For the long form, we therefore concluded that the presence of DIF did not undermine the validity of the SAHL-D as a measure of health literacy. However, the DIF results were useful in suggesting items for deletion of the short-form. We retained 13 items in the short form with maintaining reasonable reliability, suggesting that unbiased estimates of health literacy across gender, age and educational levels can be obtained from the short form SAHL-D. Whereas several short-form questionnaires have been developed to provide quick and easy assessment of functional health literacy, including the 3-item Chew’s Set of Brief Screening questions [39] these are mostly limited by the measurement of self-report health literacy or are specific to a certain health care context. The 13 items of the short SAHL-D enable quick and easy assessment of functional performance-based functional health literacy in clinical and research settings. In addition, Rasch modeling is an essential step before CAT, which could make the measure even quicker and easier to administer in clinical practice without having the disadvantages of self-report measurement, i.e. the risk of measuring self-efficacy (the belief in one’s capabilities to achieve a goal or outcome) instead of health literacy [40].

Another advantage of Rasch modeling is that the item and person invariance allows for the creation of an item-person map. The selected items showed sufficient variation in difficulty, meaning that the short form is still able to differentiate between participants with different levels of health literacy. However, the item-person map also showed that the SAHL-D is relatively easy for the samples examined in this study. The fact that the items are of medium difficulty suggests that the SAHL-D may need more difficult items if it were used to study a population with higher health literacy. However, since identifying low health literacy levels is a priority for most clinical applications, the need to test respondents at the high end of the scale (e.g. for selecting high performers) would be less common.

## Strengths & limitations

An important strength of this study is that we used IRT for psychometric testing and optimization, which is a theoretically more precise method that is gaining popularity in health sciences, although it is still less commonly used compared to CTT and factor analysis. Given that health literacy is conceptually an ability, choosing Rasch modelling for investigating of these properties is most suitable [9]. Knowledge of item difficulty and person ability is very relevant for health literacy measurement, as it enables tailoring of information to an individual’s ability levels. The fact that the SAHL-D has been administered face-to-face and that CAT would be self-administered may change item properties and this needs to be checked. We only analyzed the comprehension part, and not the recognition test, which may limit the definition of health literacy but this is due the online self-administration of CAT. Self-administration is a promising avenue since it may reduce the potential stigma that is associated with low literacy [41]. Because both the short and long forms had good psychometric properties, they can both be used to assess health literacy in scientific research. The total scores are reliable estimates of the health literacy level of a person. Currently, we are evaluating the short form, which is developed in this study, using online administration.

## Additional files

Additional file 1: SAHL-D data. Item measures by demographic groups (*N* = 1231) in order of difficulty. (DOCX 20 kb)

Additional file 2: SAHL-D data. Item measures by study sample (*N* = 1231) in order of item difficulty. (DOCX 17 kb)

Additional file 3: SAHL-D data. DIF per study sample (*N* = 1231). (DOCX 19 kb)

## Data Availability

The datasets generated and analyzed during the current study are available from the corresponding author upon reasonable request.

## Abbreviations

CAT: Computerized Adaptive Testing
CTT: Classical Test Theory
DIF: Differential Item Functioning
IRT: Item Response Theory
ISCED: International Standard Classification of Education
ISO: International Organization for Standardization
NIVEL: Netherlands Institute for Health Service Research
NVS: Newest Vital Sign
PCA: Principal Components Analysis
REALM: Rapid Estimate of Adult Literacy in Medicine
SAHL: Short Assessment of Health Literacy
TOFHLA: Test of Functional Health Literacy
ZTSD: Standardized *t* scores
