# Supplementary material for: Validation of the short assessment of health literacy (SAHL-D) and short-form development: Rasch analysis
Source: BMC Med Res Methodol. 2019 Jun 14;19:122. doi: 10.1186/s12874-019-0762-4 (PMC6567391; doi:10.1186/s12874-019-0762-4)
Supplement: Supplementary file 1 — SAHL-D data. Item measures by demographic groups (N = 1231) in order of difficulty. (DOCX 20 kb) [file 12874_2019_762_MOESM1_ESM.docx]

**Additional file 1**

| **Table 1. Item measures by demographic groups (*N* = 1231^a^) in order of difficulty** | | | | | | | |
| --- | --- | --- | --- | --- | --- | --- | --- |
|  | **Measure** | | | | | | |
| **Item** | **Male**  **(*n* = 592)** | **Female**  **(*n* = 605)** | **Age < 65**  **(*n* = 614)** | **Age ≥ 65**  **(*n* = 583)** | **Low education**  **(*n* = 265)** | **Middle education**  **(*n =* 407)** | **High education**  **(*n =* 511)** |
| Ventricle | 2.95 | 2.95 | 2.76 | 3.17 | 2.93 | 3.04 | 2.92 |
| Manic | 2.2 | 2.2 | 2.04 | 2.36 | 2.49 | 2.33 | 1.99 |
| Reflux | 2.12 | 1.59 | 1.69 | 2.01 | 1.91 | 1.93 | 1.76 |
| Gelling agent | 1.64 | 1.97 | 1.92 | 1.7 | 1.66 | 1.74 | 1.97 |
| Palliation | 1.43 | 1.67 | 1.64 | 1.46 | 1.68 | 1.9 | 1.15 |
| Pessary | 1.35 | 1.19 | 1.18 | 1.35 | 1.4 | 1.12 | 1.33 |
| Orthodontia | 1.02 | 0.95 | 0.71 | 1.25 | 0.99 | 0.76 | 1.19 |
| Hemophilia | 1.44 | 1.37 | 1.64 | 1.17 | 1.28 | 1.3 | 1.54 |
| Chlamydia | 1.03 | 0.55 | 0.6 | 1 | 0.65 | 0.56 | 1.15 |
| Malaise | 1.11 | 0.97 | 1.15 | 0.95 | 0.65 | 1.05 | 1.28 |
| Echography | 0.41 | 0.8 | 0.36 | 0.82 | 0.38 | 0.6 | 0.78 |
| Resistance | 0.23 | 1.15 | 0.65 | 0.74 | 0.88 | 0.74 | 0.51 |
| Beta blocker | 0.96 | 0.77 | 1.07 | 0.66 | 0.57 | 0.91 | 1.02 |
| Prenatal | 0.77 | 0.71 | 0.83 | 0.65 | 1.13 | 0.93 | 0.17 |
| Pancreas | 0.61 | 0.61 | 0.72 | 0.49 | 1.15 | 0.56 | 0.24 |
| Chiropractor | 0.04 | 0.24 | 0.07 | 0.2 | 0.09 | 0.14 | 0.14 |
| Psoriasis | 0.09 | -0.11 | 0.09 | -0.09 | -0.26 | -0.27 | 0.45 |
| Delirium | -0.18 | 0.24 | 0.21 | -0.16 | -0.43 | 0.26 | 0.1 |
| Apathy | 0.18 | 0.55 | 0.87 | -0.22 | 0.32 | 0.58 | 0.04 |
| Biopsy | -0.62 | -0.93 | -1.01 | -0.55 | -0.59 | -0.95 | -0.75 |
| Euphoria | -1.26 | -0.42 | -1.1 | -0.61 | -0.28 | -1.03 | -1.45 |
| Hospice | -0.26 | -0.93 | -0.32 | -0.77 | -0.51 | -0.71 | -0.31 |

| Edema | -0.16 | -0.6 | 0.04 | -0.79 | -0.46 | -0.25 | -0.35 |
| --- | --- | --- | --- | --- | --- | --- | --- |
| Obesity | -1.03 | -1.26 | -1.42 | -0.9 | -1.27 | -1 | -1.15 |
| Flaking | -1.05 | -1.29 | -1.42 | -0.94 | -1.1 | -1.41 | -0.95 |
| Apnea | -1.09 | -1.48 | -1.58 | -1 | -1.78 | -1.16 | -0.77 |
| Schizophrenia | -1.18 | -1.51 | -1.68 | -1.05 | -1.1 | -1.37 | -1.62 |
| Oncology | -0.91 | -1 | -0.84 | -1.07 | -0.76 | -0.86 | -1.29 |
| Plaque | -1.26 | -0.95 | -1.08 | -1.16 | -1 | -1.23 | -1.15 |
| Adrenalin | -2.04 | -1.72 | -2.07 | -1.75 | -2.12 | -2.11 | -1.45 |
| Achilles tendon | -2.32 | -2.61 | -2.37 | -2.5 | -2.34 | -2.92 | -2.08 |
| Defibrillation | -2.92 | -2.95 | -2.92 | -2.92 | -2.97 | -2.59 | -3.4 |
| Spinal cord lesion | -3.12 | -3.54 | -3.4 | -3.18 | -3.55 | -3.06 | -2.96 |
| ^a^ Numbers may not add up to 100% due to missing values for educational level (*n* = 14). | | | | | | | |
