# Supplementary material for: Validation of the short assessment of health literacy (SAHL-D) and short-form development: Rasch analysis
Source: BMC Med Res Methodol. 2019 Jun 14;19:122. doi: 10.1186/s12874-019-0762-4 (PMC6567391; doi:10.1186/s12874-019-0762-4)
Supplement: Supplementary file 2 — SAHL-D data. Item measures by study sample (N = 1231) in order of item difficulty. (DOCX 17 kb) [file 12874_2019_762_MOESM2_ESM.docx]

**Additional file 2**

| **Table 2 Item measures by study sample (*N* = 1231^a^) in order of item difficulty** | | | | |
| --- | --- | --- | --- | --- |
| . | **Measure** | | | |
| **Item** | **Sample 1 (*n*=541)** | **Sample 2 (*n*=223)** | **Sample 3 (*n*=323)** | **Sample 4 (*n*=110)** |
| Ventricle | 2.95 | 2.92 | 3.06 | 2.5 |
| Manic | 2.29 | 2.42 | 2.06 | 1.75 |
| Reflux | 2 | 2.03 | 1.64 | 1.4 |
| Gelling agent | 1.92 | 1.93 | 1.5 | 2.05 |
| Palliation | 1.77 | 1.01 | 1.32 | 2.27 |
| Hemophilia | 1.41 | 1.16 | 1.54 | 1.55 |
| Pessary | 1.27 | 1.16 | 1.35 | 1.31 |
| Orthodontia | 1.21 | 1.39 | 0.38 | 0.68 |
| Chlamydia | 0.97 | 0.9 | 0.62 | 0.39 |
| Malaise | 0.92 | 1.11 | 1.13 | 1.26 |
| Beta blocker | 0.85 | 0.8 | 1.04 | 0.64 |
| Prenatal | 0.7 | 0.69 | 0.74 | 0.97 |
| Pancreas | 0.61 | 0.46 | 0.64 | 0.83 |
| Resistance | 0.61 | 0.66 | 0.97 | 0.44 |
| Echography | 0.57 | 0.55 | 0.89 | 0.07 |
| Chiropractor | 0.01 | 0.14 | 0.54 | -0.27 |
| Apathy | -0.08 | 0.03 | 0.7 | 1.7 |
| Psoriasis | -0.14 | 0.16 | 0.11 | 0 |
| Delirium | -0.19 | -0.22 | 0.47 | 0.18 |
| Hospice | -0.54 | -0.62 | -0.71 | -0.16 |
| Edema | -0.64 | -0.62 | 0.06 | 0.18 |
| Euphoria | -0.65 | -0.62 | -1.89 | -0.67 |
| Biopsy | -0.75 | -0.53 | -0.95 | -0.89 |
| Oncology | -0.8 | -1.17 | -1.37 | -0.67 |
| Flaking | -0.82 | -1.06 | -1.69 | -2.27 |
| Apnea | -1.04 | -1.36 | -1.25 | -2.09 |
| Obesity | -1.22 | -0.95 | -1.06 | -1.23 |
| Plaque | -1.25 | -1.06 | -0.95 | -1.05 |
| Schizophrenia | -1.32 | -1.01 | -1.52 | -1.66 |
| Achilles tendon | -2.36 | -2.36 | -2.87 | -2.47 |
| Adrenalin | -2.42 | -1.17 | -1.52 | -2.47 |
| Spinal cord lesion | -3.21 | -3.2 | -5.50> | -2.72 |
| Defibrillation | -3.3 | -2.6 | -2.87 | -2.47 |
| ^a^ Numbers may not add up to 100% due to missing values. | | | | |
