# Supplementary material for: Validation of the short assessment of health literacy (SAHL-D) and short-form development: Rasch analysis
Source: BMC Med Res Methodol. 2019 Jun 14;19:122. doi: 10.1186/s12874-019-0762-4 (PMC6567391; doi:10.1186/s12874-019-0762-4)
Supplement: Supplementary file 3 — SAHL-D data. DIF per study sample (N = 1231). (DOCX 19 kb) [file 12874_2019_762_MOESM3_ESM.docx]

**Additional file 3**

| **Table 3. DIF per study sample *(N* = 1231)** | | | | |
| --- | --- | --- | --- | --- |
| Item | Study sample | | DIF contrast | Prob (Rasch-Welch t |
| Adrenalin | 1 | 3 | -0.9 | 0.0153 |
| Adrenalin | 2 | 4 | 1.3 | 0.0153 |
| Adrenalin | 2 | 1 | 1.24 | 0.0003 |
| Apathy | 1 | 3 | -0.78 | 0 |
| Apathy | 1 | 4 | -1.78 | 0 |
| Apathy | 3 | 2 | 0.67 | 0.0038 |
| Apathy | 3 | 4 | -0.99 | 0.0002 |
| Apnea | 1 | 4 | 1.05 | 0.0176 |
| Apnea | 2 | 4 | 0.73 | 0.1279 |
| Chiropractor | 1 | 3 | -0.53 | 0.0054 |
| Chiropractor | 3 | 4 | 0.81 | 0.0052 |
| Chlamydia | 1 | 4 | 0.58 | 0.0208 |
| Chlamydia | 2 | 4 | 0.52 | 0.0643 |
| Defibrillation | 1 | 2 | -0.7 | 0.1416 |
| Delirium | 1 | 3 | -0.66 | 0.0007 |
| Delirium | 3 | 2 | 0.69 | 0.0047 |
| Echography | 3 | 4 | 0.82 | 0.0027 |
| Edema | 1 | 3 | -0.69 | 0.0013 |
| Edema | 1 | 4 | -0.81 | 0.0029 |
| Edema | 3 | 2 | 0.68 | 0.0112 |
| Euphoria | 1 | 3 | 1.24 | 0.0006 |
| Euphoria | 2 | 3 | 1.27 | 0.0012 |
| Euphoria | 3 | 4 | -1.22 | 0.0041 |
| Flaking | 1 | 4 | 1.45 | 0.002 |
| Flaking | 1 | 3 | 0.87 | 0.0098 |
| Flaking | 2 | 4 | 1.21 | 0.0157 |
| Gelling agent | 3 | 4 | -0.56 | 0.0352 |
| Manic | 1 | 4 | 0.55 | 0.0274 |
| Manic | 2 | 4 | 0.67 | 0.0162 |
| Orthodontia | 1 | 3 | 0.83 | 0 |
| Orthodontia | 1 | 4 | 0.52 | 0.0331 |
| Orthodontia | 2 | 3 | 1.01 | 0 |
| Orthodontia | 2 | 4 | 0.7 | 0.0103 |
| Palliation | 1 | 2 | 0.76 | 0.0001 |
| Palliation | 3 | 4 | -0.96 | 0.0005 |
| Reflux | 1 | 4 | 0.59 | 0.0147 |
| Reflux | 2 | 4 | 0.63 | 0.0215 |
| Resistance | 3 | 4 | 0.53 | 0.0442 |
| Ventricle | 3 | 4 | 0.56 | 0.0452 |
